# Shelby Medical College

**Published:** 1858-09

**Authors:** 


### RUSH MEDICAL COLLEGE.

### SHELBY MEDICAL COLLEGE.

We have received the announcement of a new institution with the above title in Nashville, Tennessee. Judging from the statements made by the Dean, the faculty have every facility for instruction. Its faculty consists of

John Frederick May, M.D., Prof. of Surgery, etc.
E. B. Haskins, M.D., Prof. of Theory and Practice.
John P. Ford, M.D., Prof. of Obstetrics, etc.
Thos. L. Maddin, M.D., Prof. of Anatomy.
John H. Callender, M.D., Prof. of Materia Medica, etc.
Richard O. Curry, M.D., Prof. of Chemistry, etc.
Daniel F. Wright, M.D., Prof. of Physiology and Pathology.
H. M. Compton, M.D., Demonstrator of Anatomy.

